# Combating COVID-19 Using Generative Adversarial Networks and Artificial Intelligence for Medical Images: Scoping Review

**DOI:** 10.2196/37365

**Published:** 2022-06-29

**Authors:** Hazrat Ali, Zubair Shah

**Affiliations:** 1 College of Science and Engineering Hamad Bin Khalifa University Doha Qatar

**Keywords:** augmentation, artificial intelligence, COVID-19, diagnosis, generative adversarial networks, diagnostic, lung image, imaging, data augmentation, X-ray, CT scan, data scarcity, image data, neural network, clinical informatics

## Abstract

**Background:**

Research on the diagnosis of COVID-19 using lung images is limited by the scarcity of imaging data. Generative adversarial networks (GANs) are popular for synthesis and data augmentation. GANs have been explored for data augmentation to enhance the performance of artificial intelligence (AI) methods for the diagnosis of COVID-19 within lung computed tomography (CT) and X-ray images. However, the role of GANs in overcoming data scarcity for COVID-19 is not well understood.

**Objective:**

This review presents a comprehensive study on the role of GANs in addressing the challenges related to COVID-19 data scarcity and diagnosis. It is the first review that summarizes different GAN methods and lung imaging data sets for COVID-19. It attempts to answer the questions related to applications of GANs, popular GAN architectures, frequently used image modalities, and the availability of source code.

**Methods:**

A search was conducted on 5 databases, namely PubMed, IEEEXplore, Association for Computing Machinery (ACM) Digital Library, Scopus, and Google Scholar. The search was conducted from October 11-13, 2021. The search was conducted using intervention keywords, such as “generative adversarial networks” and “GANs,” and application keywords, such as “COVID-19” and “coronavirus.” The review was performed following the Preferred Reporting Items for Systematic Reviews and Meta-Analyses Extension for Scoping Reviews (PRISMA-ScR) guidelines for systematic and scoping reviews. Only those studies were included that reported GAN-based methods for analyzing chest X-ray images, chest CT images, and chest ultrasound images. Any studies that used deep learning methods but did not use GANs were excluded. No restrictions were imposed on the country of publication, study design, or outcomes. Only those studies that were in English and were published from 2020 to 2022 were included. No studies before 2020 were included.

**Results:**

This review included 57 full-text studies that reported the use of GANs for different applications in COVID-19 lung imaging data. Most of the studies (n=42, 74%) used GANs for data augmentation to enhance the performance of AI techniques for COVID-19 diagnosis. Other popular applications of GANs were segmentation of lungs and superresolution of lung images. The cycleGAN and the conditional GAN were the most commonly used architectures, used in 9 studies each. In addition, 29 (51%) studies used chest X-ray images, while 21 (37%) studies used CT images for the training of GANs. For the majority of the studies (n=47, 82%), the experiments were conducted and results were reported using publicly available data. A secondary evaluation of the results by radiologists/clinicians was reported by only 2 (4%) studies.

**Conclusions:**

Studies have shown that GANs have great potential to address the data scarcity challenge for lung images in COVID-19. Data synthesized with GANs have been helpful to improve the training of the convolutional neural network (CNN) models trained for the diagnosis of COVID-19. In addition, GANs have also contributed to enhancing the CNNs’ performance through the superresolution of the images and segmentation. This review also identified key limitations of the potential transformation of GAN-based methods in clinical applications.

## Introduction

### Background

In December 2019, COVID-19 broke out and spread at an unprecedented rate, given the highly contagious nature of the virus. As a result, the World Health Organization (WHO) declared it a global pandemic in March 2020 [1]. Therefore, a response to combat the spread through speedy diagnosis became the most critical need of the time. A common method for diagnosing COVID-19 is the use of a real-time reverse transcription–polymerase chain reaction (RT-PCR) test. However, with the increasing number of cases worldwide, the health care sector was overloaded as it became challenging to cope with the requirements of the tests with the available testing facilities. In addition, research has shown that RT-PCR may result in false negatives or fluctuating results [2]. Hence, diagnosis through computed tomography (CT) and X-ray images of lungs may supplement performance. Motivated by this need, alternative methods, such as automatic diagnosis of COVID-19 from lung images, were explored and encouraged. In this regard, it is well understood that artificial intelligence (AI) techniques could help inspect chest CTs and X-rays within seconds and augment the public health care sector. The use of properly trained AI models for diagnosis of COVID-19 is promising for scaling up the capacity and accelerating the process as computers are, in general, faster than humans in computations.

Many AI and medical imaging methods were explored to provide support in the early diagnosis of COVID-19, for example, AI for COVID-19 [3-5], machine learning for COVID-19 [6], and data science for COVID-19 [7]. However, AI techniques rely on large data. For example, training a convolutional neural network (CNN) to perform classification of COVID-19 versus normal chest X-ray images requires training of the CNN with a large number of chest X-ray images both for COVID-19 and for normal cases. Since the diagnosis of COVID-19 requires studying of lung CT or X-ray images, the availability of lung imaging data is vital to develop medical imaging methods. However, the lack of data on COVID-19 hampered the initial progress in developing these methods to combat COVID-19.

Many early attempts were made to collect imaging data for lungs infected with COVID-19—specifically CT and X-ray images either through a private collection in hospitals or through crowdsourcing using public platforms. In parallel, many studies have explored the use of generative adversarial networks (GANs) to generate synthetic imaging data that can improve the training of AI models to diagnose COVID-19.

GANs are a family of deep learning models that consist of 2 neural networks trained in an adversarial fashion [8-15]. The 2 neural networks, namely the generator and the discriminator, attempt to minimize their losses, while maximizing the loss of the other. This training mechanism improves the overall learning task of the GAN model, particularly for generating data. GANs have recently been studied for computer vision and medical imaging tasks, such as image generation, superresolution, and segmentation [9,10]. Given the significant potential of GANs in medical imaging, it was intuitive that many researchers were tempted to explore the use of GANs for data augmentation of imaging data on COVID-19. In addition, some researchers also used GANs for segmentation and superresolution of lung images.

This scoping review focuses on providing a comprehensive review of the GAN-based methods used to combat COVID-19. Specifically, it covers the studies where GANs have been used for lung CT and X-ray images to diagnose COVID-19 or to enhance the performance of CNNs for the diagnosis of COVID-19 (eg, by data augmentation or superresolution).

### Research Problem

GANs have gained the attention of the medical imaging research community. As the COVID-19 pandemic continued to grow in 2020 and 2021, the research community faced a significant challenge due to the scarcity of medical imaging data on COVID-19 that can be used to train AI models (eg, CNN) to perform COVID-19 diagnosis automatically. Given the popularity of GANs for image synthesis, researchers turned to exploring the use of GANs for data augmentation of lung radiology images. Many studies were conducted to use different variants of GANs for data augmentation of lung CT images and lung X-ray images. Similarly, a few studies also used GANs for the diagnosis of COVID-19 from lung radiology images. However, to the best of our knowledge, there is no review on the role of GANs in addressing the challenges related to COVID-19 data scarcity and diagnosis. The following research questions related to COVID-19 imaging data were considered for this review:

What were the common applications of GANs proposed for challenges related to COVID-19?

- Which architectures of GANs are most commonly applied for data augmentation tasks related to COVID-19?
- Which imaging modality is the popular choice for the diagnosis of COVID-19?
- What were the most commonly used data sets of CT and X-ray images for COVID-19?
- What studies were conducted with open-source code to reproduce the results?
- What studies were conducted and presented to radiology experts for evaluation of the suitability toward future use in clinical applications?

The results of this review will be helpful for researchers and professionals in the medical imaging and health care domain who are considering using GAN-based methods to address challenges related to COVID-19 imaging data and to address the challenge in improving automatic diagnosis using radiology images.

## Methods

### Study Design

In this work, a scoping review was conducted following the Preferred Reporting Items for Systematic Reviews and Meta-Analyses Extension for Scoping Reviews (PRISMA-ScR) guidelines [16]. The methods for performing the study are described next.

### Search Strategy

#### Search Sources

A search was conducted from October 11-13, 2021. The search was performed on the following 5 databases: PubMed, IEEEXplore, Association for Computing Machinery (ACM) Digital Library, Scopus, and Google Scholar. In the case of Google Scholar, only the first 99 results were retained as the results beyond 99 items were highly irrelevant to the scope of the study. Similarly, in the case of ACM Digital Library, the first 100 results were retained as a lack of relevancy to the study was obvious in results beyond 100.

#### Search Terms

The search terms used in this study were chosen from the literature with guidance from experts in the field. The terms were chosen based on the intervention (eg, “generative adversarial networks,” “GANs,” “cycleGANs”) and the target application (eg, “COVID-“19”, “coronavirus,” “corona pandemic”). The exact search strings used in the search for this study are available in Multimedia Appendix 1.

### Search Eligibility Criteria

This study focused on the applications of GANs in analyzing radiology images of lungs for COVID-19, used for any purpose such as data augmentation or synthesis, diagnosis, superresolution, and prognosis. Only those studies were included that reported GAN-based methods for analyzing chest X-ray images, chest CT images, and chest ultrasound images. Studies that reported GAN-based methods for analyzing nonlung images were removed. Any studies that used deep learning methods but did not use GANs were also excluded. Studies reporting GANs for nonimaging data were also excluded. To provide a list of reliable studies, only peer-reviewed articles, conference papers, and book chapters were included. Preprints, conference abstracts, short letters, and commentaries were excluded. Similarly, review articles were also excluded. No restrictions were imposed on the country of publication, study design, or outcomes. Studies that were written in English and were published from 2020 to 2022 were included. No studies before 2020 were included.

### Study Selection

Two reviewers (authors HA and ZS) screened the titles and abstracts of the search results. Initial screening by the 2 reviewers was performed independently. Disagreement occurred for only 9 articles. The disagreement was resolved through mutual discussion and consensus. For measuring the disagreement, Cohen κ [17] was calculated to be 0.89, which shows good agreement between the 2 independent reviewers. Multimedia Appendix 2 shows the matrix for the agreement between the 2 independent reviewers.

### Data Extraction

Multimedia Appendix 3 shows the form for extraction of the key characteristics. The form was pilot-tested and refined in 2 rounds, first by data extraction for 5 studies and then by data extraction for another 5 studies. This refinement of the form ensured that only relevant data were extracted from the studies. The 2 reviewers (HA and ZS) extracted the data from the included studies, related to the GAN-based method, applications, and data sets. Any disagreement between the reviewers was resolved through mutual consensus and discussions. As the disagreements at the study selection stage were resolved through careful and lengthy discussions, the disagreement at the data extraction was only minor.

### Data Synthesis

After extraction of the data from the full text of the identified studies, a narrative approach was used to synthesize the data. The use of GAN-based methods was classified in terms of the application of GANs (eg, augmentation, segmentation of lungs); the type of GAN architecture, if reported (eg, conditional GAN or cycleGAN); and the modality of the imaging data for which the GAN was used (eg, CT or X-ray imaging). Similarly, the studies were classified based on the availability of the data set (eg, public or private), the size of the data set (eg, the number of images in the original images and the number of images after augmentation with the GAN, if applicable), and the proportion of the training and test sets as well as the type of cross-validation. The data synthesis was managed and performed using Microsoft Excel.

## Results

### Search Results

**Figure 1:**
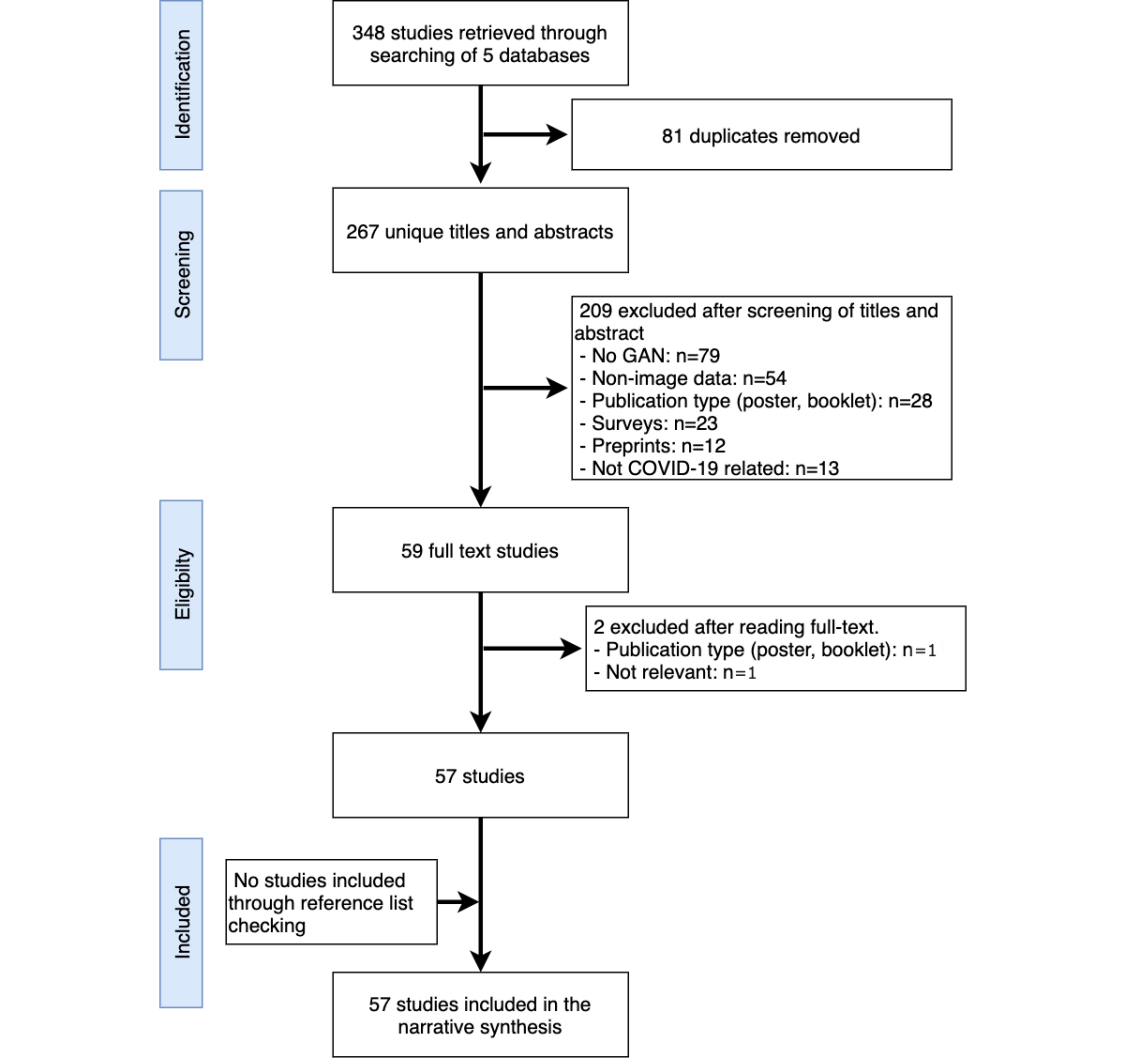
PRISMA-ScR flowchart for the search outcomes and selection of studies. GAN: generative adversarial network; PRISMA-ScR: Preferred Reporting Items for Systematic Reviews and Meta-Analyses Extension for Scoping Reviews.

From 5 online databases, a total of 348 studies were retrieved (see Figure 1). Of the 348 studies, 81 (23.3%) duplicates were removed. The titles and abstracts of the remaining 267 (76.7%) studies were carefully screened as per the criteria of inclusion and exclusion. The screening of the titles and abstracts resulted in the exclusion of 208 (77.9%) studies (see Figure 1 for reasons of exclusion). After the full-text reading of the remaining 59 (22.1%) studies, 2 (3%) studies were excluded following the inclusion/exclusion criteria. Finally, a total of 57 (97%) studies were included in this review. No additional studies were found through reference list checking. As per the yearwise publication, 15 (26%) of 57 studies were published in 2020 and 41 (72%) of 57 were published in 2021.

### Demographics of the Included Studies

**Figure 2:**
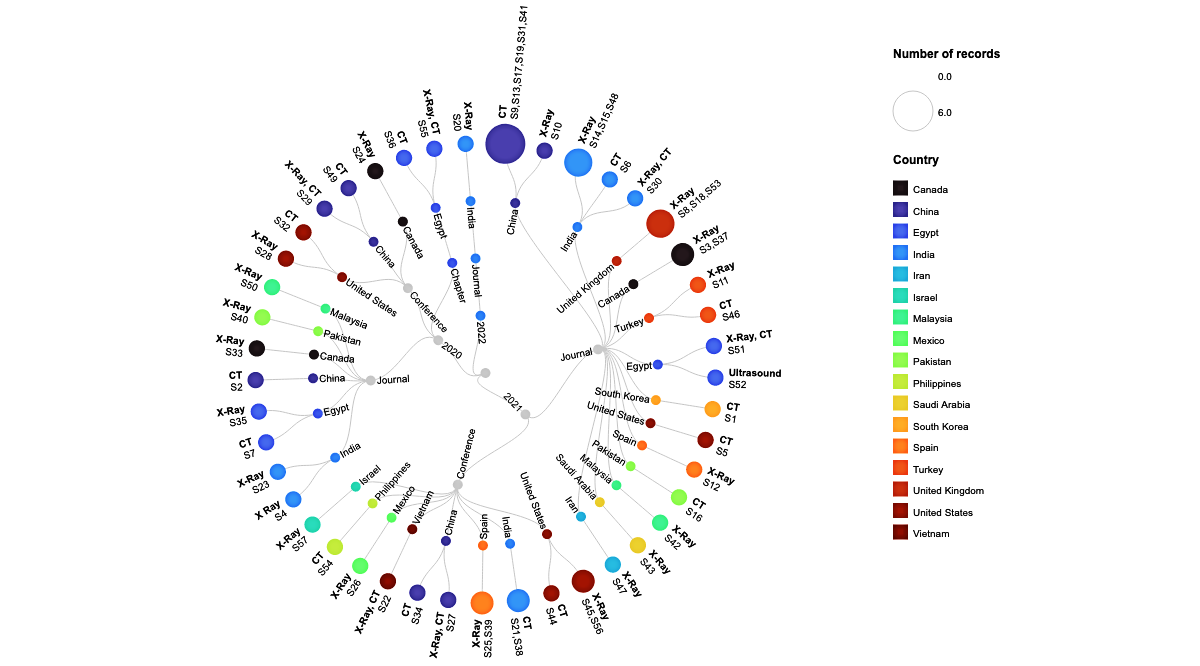
Characteristics of the included studies showing the publication type, country of publication, and modality of data. The number of studies is reflected by the size of the terminal node. The numbers S1-S57 refer to the included studies. CT: computed tomography.

Among the included studies (N=57), 37 (65%) studies were published articles in peer-reviewed journals, 18 (32%) studies were published in conference proceedings, and 2 (4%) studies were published as book chapters. No thesis publication was found relevant to the scope of this review. Around one-fourth of the studies (n=15, 26%) were published in 2020. Most of the studies were published in 2021 (n=41, 72%). The included studies were published in 14 countries. The largest number of publications were from China (n=12, 21%), followed by India (n=10, 18%). Both the United States and Egypt published the same number of studies (n=6, 11%, each). The characteristics are summarized in Table 1 and Multimedia Appendix 4. Figure 2 (see [18-74]) shows the demographics of the included studies, along with the modality of the chest images used.

**Table 1:** Characteristics of the included studies (N=57). Demographics are shown for type of publication, country of publication, and year of publication.

Characteristics		Studies, n (%)
**Publication type**		
	Journal	37 (65)
	Conference	18 (32)
	Book chapter	2 (4)
**Country**		
	China	12 (21)
	India	10 (18)
	United States	6 (11)
	Egypt	6 (11)
	Canada	4 (7)
	Spain	3 (5)
	Malaysia	2 (4)
	Turkey	2 (4)
	Pakistan	2 (4)
	Vietnam	1 (2)
	Mexico	1 (2)
	South Korea	1 (2)
	Philippines	1 (2)
	Israel	1 (2)
**Year of publication**		
	2020	15 (26)
	2021	41 (72)
	2022	1 (2)

### Application of the Studies

As shown in Table 2, the included studies have reported 5 different tasks being addressed: augmentation (data augmentation), diagnosis of COVID-19, prognosis, segmentation (to identify the lung region), and diagnosis of lung diseases. As the diagnosis of COVID-19 using medical imaging has been a priority since the pandemic started, 39 (68%) of 57 studies reported the diagnosis of COVID-19 as the main focus of their work [19-21, 23-33, 35-37, 39, 41, 42, 44, 46, 50, 52, 53, 55, 56, 58-60, 63-69, 71, 72]. In addition, 9 (16%) studies reported data augmentation as the main task addressed in the work [18,43,45,49,54,61,62], 1 (2%) study reported prognosis of COVID-19 [22], 3 (5%) studies reported segmentation of lungs [34,51,57], and 1 (2%) study reported diagnosis of multiple lung diseases [47].

**Table 2:** Applications of using GAN^a^-based methods and types of GANs.

Applications		Studies (N=57), n (%)
**Applications addressed in the studies**		
	Diagnosis	39 (68)
	Data augmentation	9 (16)
	Segmentation+diagnosis	3 (5)
	Segmentation	3 (5)
	Diagnosis of lung disease	1 (2)
	Prognosis	1 (2)
	Prognosis+diagnosis	1 (2)
**Applications of using GANs**		
	Augmentation	42 (74)
	Diagnosis	5 (9)
	Superresolution	3 (5)
	Segmentation	3 (5)
	Feature extraction	2 (4)
	Prognosis	1 (2)
	3D synthesis	1 (2)
**Type of GAN used**		
	GAN	17 (30)
	CycleGAN	9 (16)
	Conditional GAN	9 (16)
	Deep convolutional GAN	4 (7)
	Auxiliary classifier GAN	4 (7)
	Superresolution GAN	2 (4)
	3D conditional GAN	2 (4)
	BiGAN	1 (2)
	Random GAN	1 (2)
	Pix2pix GAN	1 (2)

^a^GAN: generative adversarial network.

**Figure 3:**
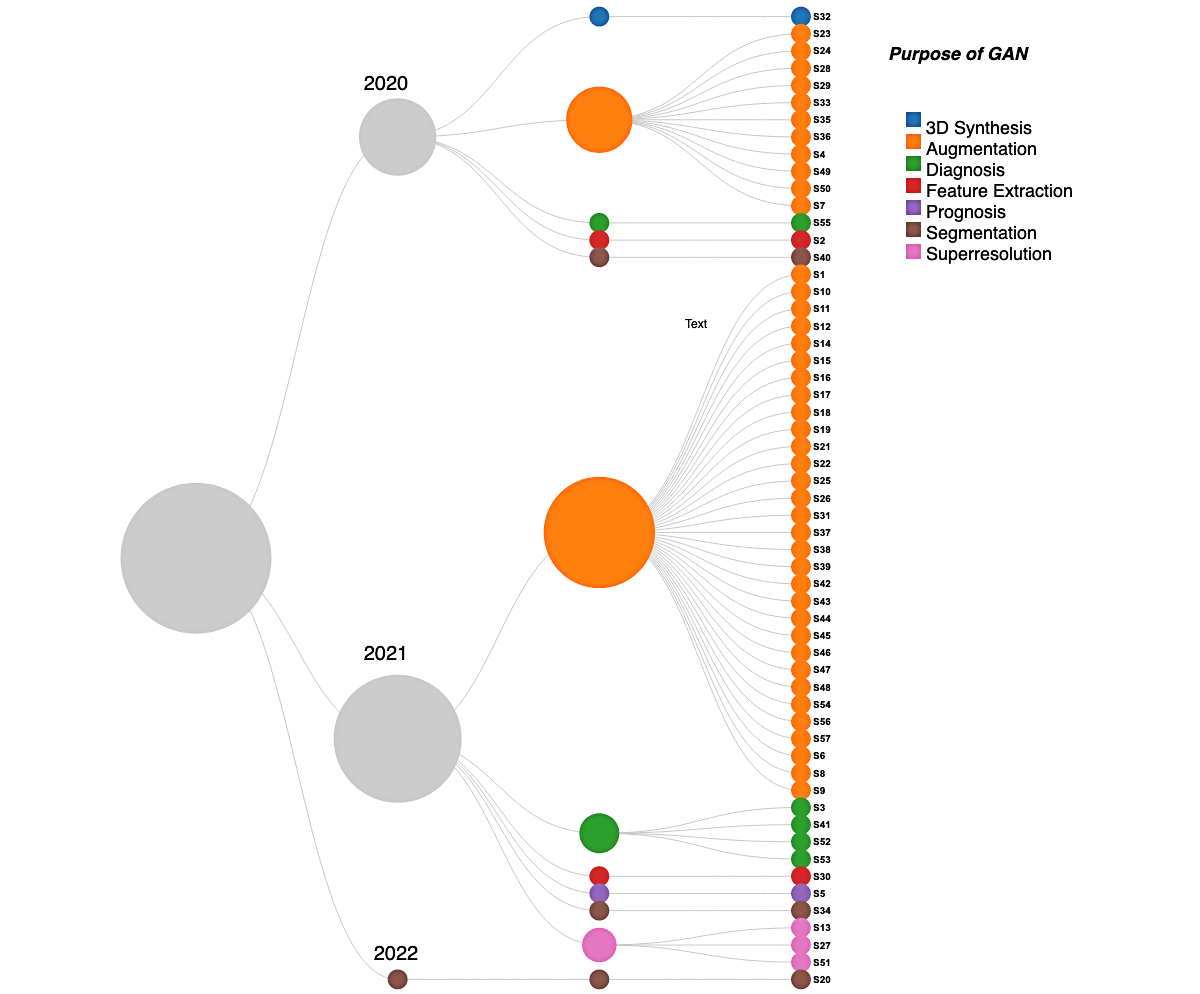
Major applications of GANs in the included studies. The number of publications for each application is reflected by the size of the circle in the second-last layer. The numbers S1-S57 refer to the included studies. GAN: generative adversarial network.

The majority of the studies used GANs to augment the data, where they reported the use of GANs to increase the data set size. Specifically, 42 (74%) studies used GAN-based methods for data augmentation [18, 21, 23-29, 31-36, 38-43, 45, 46, 48, 50, 52-56, 59-67, 71, 73, 74]. The augmented data were then used to improve the training of different CNNs to diagnose COVID-19. In addition, 3 (5%) studies used GANs for segmentation of the lung region within the chest radiology images [37,51,57], 3 (5%) studies used GANs for superresolution to improve the quality of the images before using them for diagnosis purposes [30,44,68], 5 (9%) studies used GANs for the diagnosis of COVID-19 [20,58,69,70,72], 2 (4%) studies used GANs for feature extraction from images [19,47], and 1 (2%) study used a GAN-based method for prognosis of COVID-19 [22]. The prevalent mode of imaging is the use of 2D imaging data, and 1 (2%) study reported a GAN-based method for synthesizing 3D data [49]. Figure 3 (see [18-74]) shows the mapping of the applications of GAN-based methods for all the included studies.

Different variants have been proposed for GAN architectures since their inception. The most common type of GAN used in these studies was the cycleGAN, used in 9 (16%) studies [29,35,36,42,46,54,56,70,74]. The cycleGAN is an image translation GAN that does not require paired data to transform images from one domain to another. Other popular types of GANs were conditional GAN used by 9 (16%) studies [18,22,24,25,33,37,41,57,60], deep convolutional GAN used by 4 (7%) studies [21,38,43,67], and auxiliary classifier GAN used by 4 (7%) studies [32,40,55,69]. The superresolution GAN was used by 2 (4%) studies [44,68], and 1 (2%) study reported the use of multiple GANs, namely Wassertein GAN, auxiliary classifier GAN, and deep convolutional GAN, and compared their performances for improving the quality of images [31].

Of the 57 studies, only 10 (18%) [18,19,26,27,30,34,43,61-73] reported changes to the architecture of the GAN they were using. In the rest of the studies, no major changes were reported to the architecture of the GAN.

### Characteristics of the Data Sets

The included studies applied GANs on lung radiology images obtained using various modalities. Specifically, the use of X-ray images dominated the studies. In total, 29 (51%) studies used X-ray images of lungs [20,21, 25, 27-29, 31, 32, 35, 37, 40-43, 45, 50, 52, 54, 56, 57, 59, 60, 62, 64, 65, 67, 70, 73, 74], while 21 (37%) studies used CT images [18,19,22-24,26,30,33,34,36,38,48,49,51,53,55,58,61,63,66,71], and 6 (11%) studies reported the use of both X-ray and CT images [39,44,46,47,68,72]. Only 1 (2%) study used ultrasound images for COVID-19 diagnosis [69], which shows that ultrasound is not a popular imaging modality for training GANs and other deep learning models for COVID-19 detection (also see Figure 4). Of the 57 studies, most (n=47, 82%) used image data sets that are publicly available. In 10 (18%) studies, the data sets used are private. Table 3 provides a list of the various data sets used in the included studies and whether they are publicly available data sets or private. The most commonly used data set was the COVIDx data set available on Github, used by 26 (46%) studies.

**Figure 4:**
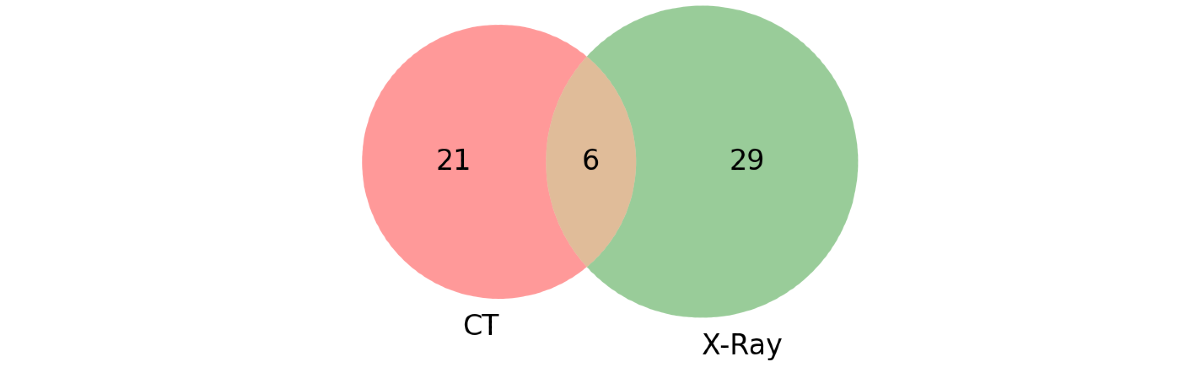
Venn diagram showing the number of studies using CT vs X-ray images. Only 1 (2%) study reported the use of ultrasound images (not reflected here). CT: computed tomography.

**Table 3:** Resources of the data sets used in the included studies. The name is provided only if available.

Platform (name)	Public or private	Modality of imaging
Kaggle	Public [75]	CT^a^
Github	Public [76]	CT
Github	Public [77]	CT
Github (Covidx)	Public [78]	X-ray, CT
Github	Public [79]	X-ray
Kaggle (Tawsif)	Public [80]	X-ray
Github	Public [81]	X-ray
Kaggle	Public [82]	X-ray
Mendeley	Public [83]	CT
Website	Public [84]	CT
Kaggle (Allen Institute)	Public [85]	CT
Kaggle (RSNA)	Public [86]	X-ray
Website	Public [87]	CT
Github	Public [88]	Ultrasound
Kaggle	Public [89]	X-ray
Website (Italian Society of Medical and Interventional Radiology)	Public [90]	X-ray
First Affiliated Hospital of the University of Science and Technology China	Private	CT
Massachusetts General Hospital, Brigham and Women's Hospital	Private	CT
Comlejo Hospitalario Universitario de A Coruna Spain	Private	X-ray

^a^CT: computed tomography.

The majority of the studies reported the size of the data set in terms of the number of images. The number of images used was greater than 10,000 in only 7 (12%) studies [20,22,30,39,63,66,74], while 3 (5%) studies used images between 5000 and 10,000 [33,47,64]. The most common range for the number of images used was 1000-5000 images used in 15 (26%) studies. Around one-fifth of the studies (n=11, 19%) used between 500 and 1000 images. In 11 (19%) other studies, the number of images used was less than 500. No study reported a number of images less than 100. The maximum number of images was 84,971, used by Uemura et al [22]. Only a few of the studies reported the number of patients for whom the data were used: 1 (2%) study used data for more than 1000 patients [26], 2 (4%) studies used data for 500-1000 patients [29,42], 6 (11%) studies used data for 100-500 patients [19,22,24,30,38,71], and 4 (7%) studies used data for less than 100 patients [18,49,66,69]. The number of patients was not reported in the rest of the studies.

After augmentation using GANs, the studies increased the number of images to several thousand, with a maximum number of 21,295 [54]. In 6 (11%) studies using GANs for data augmentation, the number of images increased to more than 10,000. In 3 (5%) studies, the number of images increased to 5000-10,000. In 9 (16%) studies, the number of images increased to 1000-5000, and in 2 (4%) studies, the number of images increased between 500 and 1000. No study reported data augmentation output below 500 images.

### Evaluation Mechanisms

Generally, the popular metrics for evaluating the diagnosis and classification performances of neural networks are accuracy, precision, recall, dice score, and area under the receiver operating characteristic curve (AUROC). To evaluate the performance of neural networks for diagnosis of COVID-19, 38 (67%) of the 57 studies used accuracy, along with metrics such as precision, recall, and dice score [21,23-28,31-34,36,38,40,43-48,52,53,55,56,58-60,63-72,74]. Around one-fourth of the studies (n=18, 32%) used sensitivity and specificity. In addition, 12 (21%) studies used the AUROC [19,20,26,30,32,46-48,50,51,68,74]. The numbers do not add up, as many studies used more than 1 metric for evaluation. In addition to the metrics mentioned here, 1 (2%) study used additional metrics, namely concordance index and relative absolute error, to evaluate prognosis and survival prediction for patients with COVID-19 [22].

Likewise, the popular metrics used to assess the quality of the synthesized images are the structural similarity measure (SSIM), the peak signal-to-noise ratio (PSNR), and the Fréchet inception distance (FID). Of the 57 studies included, 6 (11%) used the SSIM [18,30,49,60-62], 5 (9%) used the PSNR [18,30,49,61,62], and 3 (5%) used the FID metric [18,43,62] for evaluation.

The majority of the studies (n=42, 74%) reported having the data split between independent training and test sets. A few of the studies (n=6, 11%) reported 5-fold or 10-fold cross-validation for training and evaluation of the model. For almost one-sixth of the studies (n=9, 16%), the information on cross-validation was not available.

### Reproducibility and Secondary Evaluation

This review also summarizes the studies in which the authors provided the implementation code. Only 7 (12%) of the 57 studies provided links for their code [19,20,34,47,48,66,70]. Only 2 (4%) studies reported a secondary evaluation by radiologists/doctors/experts by presenting the outcome of the results obtained by their models [19,45]. In addition, 1 (2%) study presented the results of end-to-end diagnosis of COVID-19 from CT images to 3 radiologists for a second opinion [19], and 1 (2%) study presented synthetic X-ray images to 2 radiologists for a second opinion on the quality of the generated X-ray images [45].

## Discussion

### Principal Findings

In this review, a significant rise in the number of studies on the topic was found in 2021 compared to 2020. This makes sense as the first half of 2020 saw only initial cases of COVID-19 infection, and research on the use of GANs for COVID-19 had yet to gain pace. Lung radiology image data for COVID-19-positive examples gradually became available during this period and increased only in the latter part of 2020. The highest number of studies were published from China and India (n=22). There can be 2 possible reasons for this. First, the 2 countries hold the top 2 spots on the ranking of the world's most populous countries. Second, the COVID-19 pandemic started in China, hence prompting earlier research efforts there.

**Figure 5:**
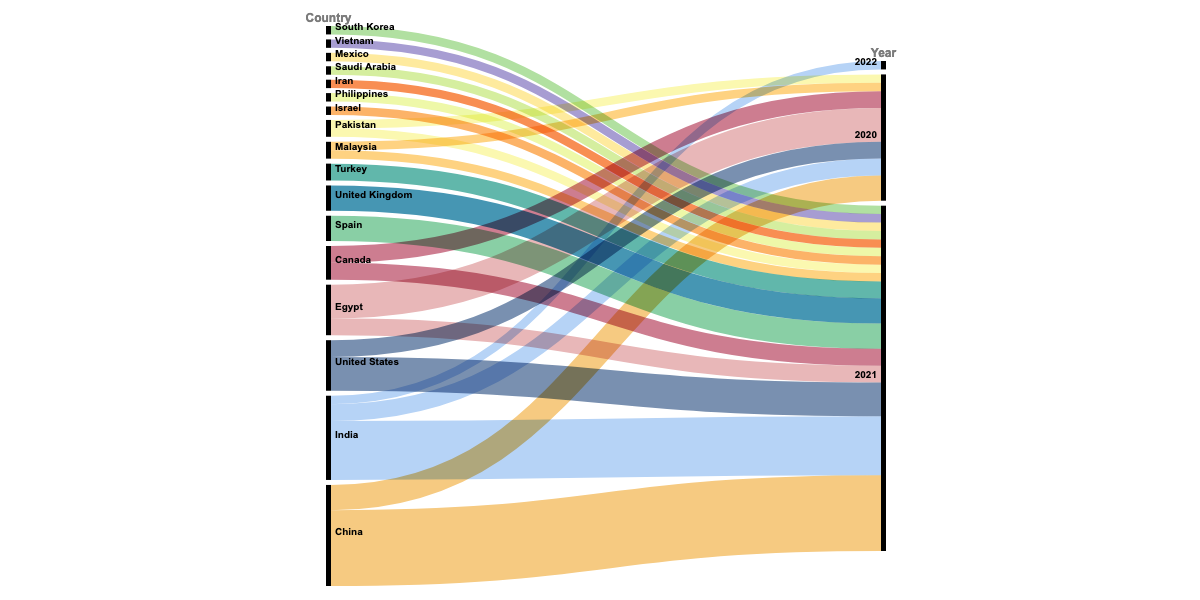
Mapping of correlation between publications from each country vs year of publication. Studies in 2020 originated mostly from China, India, Egypt, and Canada. In 2021, many other countries also contributed to the published research.

Interestingly, the same number of studies (n=6) were published from the United States and Egypt each. The correlation mapping in Figure 5 shows that most of the studies published in 2020 originated from China, India, Egypt, and Canada. However, in 2021, many other countries also contributed to the published research. The number of journal papers was twice that of conference papers. This is surprising as journal publications would typically require more time in paper processing compared to conferences. It can be possible that many authors turned to journal submissions as, during the start of the pandemic, many conferences were suspended initially before moving to the online (virtual) mode.

In the majority of the included studies (n=39), the main task was to perform diagnosis of COVID-19 using lung CT or X-ray images. In these studies, a GAN was used as a submodule of the overall framework, and diagnosis was performed with the help of variants of CNNs, such as ResNet, VGG16, and Inception-net. In the included studies, GANs were used for 7 different purposes: data augmentation, segmentation of lungs within chest radiology images, superresolution of lung images to improve the quality of the images, diagnosis of COVID-19 within the images, feature extraction, prognosis studies related to COVID-19, and synthesis of 3D volumes of CT. Around 73% of the included studies used GAN-based methods for data augmentation to address the data scarcity challenge of COVID-19. It is not unexpected, as data augmentation is the most popular application of GANs. Only 1 study used the 3D variant of GAN for 3D synthesis of CT volumes. This is not surprising as 3D synthesis of CT volumes using 3D GANs is computationally expensive. The computations for the 3D synthesis of CT volumes may exceed the available resources of the graphics processing unit (GPU).

Since there are many variants of GANs, this review also looked at the most commonly used GAN architecture in the included studies. The most common choice of GAN in the included studies was the cycleGAN used in 9 studies. The cycleGAN is a GAN architecture that comprises 2 generators and 2 discriminators and does not require pair-to-pair training data [11]. Hence, it was a popular choice to generate COVID-19–positive images from normal images.

This review analyzed the common imaging modality for the different applications related to COVID-19. As chest X-ray imaging and CT scans are the most popular imaging methods for studying the infection in individuals, the studies included in this review were those that used these 2 imaging modalities. Specifically, 35 studies used X-ray images, and 21 studies used CT images. Some of the studies (n=6) also used both CT and X-ray images for diagnosis by training different models or for the transformation of images from X-ray to CT. Though ultrasound imaging is not prevalent in the clinical diagnosis of COVID-19, 1 study reported using ultrasound images to diagnose COVID-19 with GANs. No other modality of imaging was used by the included studies.

The majority of the included studies (n=47) used data that are available publicly on Github, Kaggle, or other publicly accessible websites. These data are acquired from multiple sources (eg, collected from more than 1 hospital or through crowdsourcing), which makes them more diverse and hence more useful for training of GAN models. Similarly, it is hoped that the use of publicly accessible data will also encourage other researchers to conduct experiments on the data sets. The rise of publications in 2021 can also be linked to the availability of publicly available data sets that continued to rise as the number of COVID-19 cases continued to grow. A few of the included studies (n=10) used private or proprietary data sets, and hence, the details about those data sets are only limited to what has been described in the corresponding studies.

Only 13 studies provided information on the number of individuals whose data were used in the included studies. Among these, only 1 study used data for more than 1000 individuals [26] and 2 studies used data for more than 500 individuals [29,42]. The remaining 10 studies used data for less than 500 individuals. Given the size of the population infected with COVID-19 (418+ million as of writing this, reported from John Hopkins University Coronavirus Resource Center [91]), the need for experiments with much more extensive data is obvious. As a result of having more data, learning inherent features within the radiology images by using GANs will become more generalized with training on larger data. There is still more need to contribute to publicly accessible data.

### Practical and Research Implications

This review presented the different studies that used GANs for various COVID-19 applications. Data augmentation of COVID-19 imaging data was the most common application in the included studies. The augmented data can significantly improve the training of AI methods, particularly deep learning methods used for COVID-19 diagnosis. This review found that for most of the studies, the current CT and X-ray imaging data (even if smaller in size) are already available through publicly accessible links on Github, Kaggle, or institutional websites. This should encourage more researchers to build upon the available data sets and train more variants of deep learning and GAN-based methods to speed up the research progress on COVID-19. Similarly, researchers can also add to the existing data set on Github by uploading their data to the current data repositories. An example of crowdsourcing of data is the COVIDx image repository for lung X-ray images (see Table 3).

This review identified that the code to reproduce the results was not available for the majority of the studies. Only 7 of the included studies provided a public link to the code. Availability of a public repository to reproduce the results for diagnosis or augmented data can help in advancing the research as well as increase the trust and reliance on the reported results in terms of the quality of the generated images or the accuracy reports for the diagnosis. In addition, the reproducibility by this code was not assessed by this review, as it was beyond the scope of this review. Careful and responsible studies are needed to make an assessment of the published methods for transformation into clinical applications.

The majority of the included studies (n=43) did not provide information on the number of patients, although they did mention the number of images used in the experiments. So, it is unclear how many images were used per individual. Hence, the lack of information limits the ability of the readers to evaluate the performance in the context of the number of patients. Moreover, for public data sets with crowd-sourced contributions, it is challenging to trace back the number of images to the number of individuals.

Validation of the performance of GANs in terms of the quality/usability of the generated images has a significant role in promoting the acceptability of the methods. Of the included studies, only 2 studies reported that the results were presented to radiologists/clinicians for a secondary validation. In 1 study on the synthesis of X-ray images, the radiologists agreed that the quality of the X-rays has improved but falls short of diagnostic quality for use in clinics [45]. Although using GAN-based methods in COVID-19 is tempting for many researchers, the lack of evaluation by radiologists or using GAN-based methods without radiologists and clinicians in the loop will hinder the acceptability of these methods for clinical applications. In addition, it is beyond the scope of this review to evaluate a study based on reporting of secondary evaluation by the radiologists, though a secondary assessment by the radiologists would have added value to the studies and increased their acceptability. The lack of details related to the individuals whose COVID-19 data were used in these studies may also hinder their acceptance for transformation into clinical applications. The training of GANs is usually computationally demanding, requiring GPUs. More edge computing–based implementations are needed for clinical applications to make these models compatible for implementation on low-power devices. This will increase the acceptability of these methods in clinical devices.

### Strengths and Limitations

#### Strengths

Though several reviews can be found on the applications of AI techniques in COVID-19, no review was found that focused on the potential of GAN-based methods to combat COVID-19. Compared to other reviews [3,4,6,7] where the scope is too broad as they attempted to cover many different AI models, this review provided a comprehensive analysis of the GAN-based approaches used primarily on lung CT and X-ray images. Similarly, many reviews covered the applications of GANs in medical imaging [10,12-15]; their applications in lung images for COVID-19 have not been reviewed before. So, this review may be considered the first comprehensive review that covers all the GAN-based methods used for COVID-19 imaging data for different applications in general and data augmentation in particular. Thus, it is helpful for the readers to understand how GAN-based approaches were used to address the problem of data scarcity and how the synthetic data (generated by GANs) were used to improve the performance of CNNs for COVID-19. This review provided a thorough list of the various publicly available data sets of lung CT, lung X-ray, and lung ultrasound images. Hence, this can serve as a single point of contact for the readers to explore these data set resources and use them in their research work. This review is consistent with the PRISMA-ScR guidelines for scientific reviews [16].

#### Limitations

This review included studies from 5 databases: PubMed, IEEEXplore, ACM Digital Library, Scopus, and Google Scholar. Hence, it is possible that some literature that is not indexed in these libraries might have been left out. However, given the coverage by these popular databases, the included studies form a comprehensive representation of the applications of GANs in COVID-19. The review, for practical reasons, included studies published only in English and did not include studies in other languages. Since the scope of this review was limited to lung images only, the potential of GANs for other types of medical data, such as electronic health records, textual data, and audio data (recordings of coughing), was not covered in this review. The results and interpretations presented in this review are derived from the available information in the included studies. Since different studies may have variations and even missing details in their reporting of the data set, the training and test sets, and the validation mechanism, a direct comparison of the results might not be possible. Inconsistent information on the number of images, the training mechanism for GANs, and the selection of test set examples may have affected the findings of this review. In addition, by modern standards of training deep learning models, the size of data reported in most included studies is too small. So, the results reported in the studies in terms of diagnosis accuracy may not generalize well. The findings and the discussions of this review are mainly based on the authors’ understanding of GANs (and other AI methods) and do not necessarily reflect the comments and feedback of the doctors and clinicians.

### Conclusion

This scoping review provided a comprehensive review of 57 studies on the use of GANs for COVID-19 lung imaging data. Similar to other deep learning and AI methods, GANs have demonstrated outstanding potential in research on addressing COVID-19 diagnosis performance. However, the most significant application of GANs has been data augmentation by generating synthetic chest CT or X-ray imaging data from the existing limited-size data, as the synthetic data showed a direct bearing on the enhancement of the diagnosis. Although GAN-based methods have demonstrated great potential, their adoption in COVID-19 research is still in a stage of infancy. Notably, the transformation of GAN-based methods into clinical applications is still limited due to the limitations in the validation of the results, the generalization of the results, the lack of feedback from radiologists, and the limited explainability offered by these methods. Nevertheless, GAN-based methods can assist in the performance enhancement of COVID-19 diagnosis, even though they should not be used as independent tools. In addition, more research and advancements are needed toward the explainability and clinical transformations of these methods. This will pave the way for a broader acceptance of GAN-based methods in COVID-19 applications.

## Appendix

Multimedia Appendix 1 Search strategy.

Multimedia Appendix 2 Interrater agreement matrices for study selection steps.

Multimedia Appendix 3 Data extraction form.

Multimedia Appendix 4 Characteristics of the included studies.

## Abbreviations

ACM: Association for Computing Machinery
AI: artificial intelligence
AUROC: area under the receiver operating characteristic curve
CNN: convolutional neural network
CT: computed tomography
FID: Fréchet inception distance
GAN: generative adversarial network
GPU: graphics processing unit
PRISMA-ScR: Preferred Reporting Items for Systematic Reviews and Meta-Analyses Extension for Scoping Reviews
PSNR: peak signal-to-noise ratio
RT-PCR: reverse transcription–polymerase chain reaction
SSIM: structural similarity measure
