# Supplementary material for: Combating COVID-19 Using Generative Adversarial Networks and Artificial Intelligence for Medical Images: Scoping Review
Source: JMIR Med Inform. 2022 Jun 29;10(6):e37365. doi: 10.2196/37365 (PMC9246088; doi:10.2196/37365)
Supplement: Multimedia Appendix 1 [file medinform_v10i6e37365_app1.docx]

# Appendix 1: Search Strategy

Database(s): Pubmed, IEEE Xplore, ACM Digital Library, Scopus and Google Scholar.

Years: 2020 to 2022

Search dates: 11 October 2021 to 13 October 2021

Initial raw search results: 348

| **Database** | **Search strategy** | **Hits** |
| --- | --- | --- |
| IEEExplore | ((Generative Adversarial Networks) OR (GANs) OR (cycleGAN) OR (styleGAN)) AND ((Coronavirus) OR (COVID-19) OR (COVID19) OR (SARS-COV-2) OR (2019-nCOV) OR (CORONA PANDEMIC)) | 22 |
| ACM Digtial Library | [[Abstract: "generative adversarial networks"] OR [Abstract: "GANs"] OR [Abstract: "cycleGAN"] OR [Abstract: “styleGAN”]] AND [[Abstract: coronavirus] OR [Abstract: "covid-19"] OR [Abstract: COVID19] OR [Abstract: "sars-cov-2"] OR [Abstract: "2019-nCOV"] OR [Abstract: "corona pandemic"]] | 100 |
| Pubmed | ((Generative Adversarial Networks) OR (GANs) OR (cycleGAN) OR (styleGAN)) AND ((Coronavirus) OR (COVID-19) OR (COVID19) OR (SARS-COV-2) OR (2019-nCOV) OR (CORONA PANDEMIC)) | 51 |
| Scopus | ( ( TITLE-ABS-KEY ( "Generative Adversarial Networks" ) OR TITLE-ABS-KEY ( "GANs" ) OR TITLE-ABS-KEY ( "cycleGAN" ) OR TITLE-ABS-KEY ( "styleGAN" ) ) ) AND ( ( TITLE-ABS-KEY ( coronavirus ) ) OR ( TITLE-ABS-KEY ( covid-19 ) ) OR ( TITLE-ABS-KEY ( "covid19" ) ) OR ( TITLE-ABS-KEY ( "2019-ncov" ) ) OR ( TITLE-ABS-KEY ( "sars-cov-2" ) OR ( "corona pandemic" ) ) ) AND ( LIMIT-TO ( PUBYEAR , 2021 ) OR LIMIT-TO ( PUBYEAR , 2020 ) ) | 76 |
| Google Scholar | ((Generative Adversarial Networks) OR (GANs) OR (cycleGAN) OR (styleGAN)) AND ((Coronavirus) OR (COVID-19) OR (COVID19) OR (SARS-COV-2) OR (2019-nCOV) OR (CORONA PANDEMIC)) | 99 |
