# Supplementary material for: Combating COVID-19 Using Generative Adversarial Networks and Artificial Intelligence for Medical Images: Scoping Review
Source: JMIR Med Inform. 2022 Jun 29;10(6):e37365. doi: 10.2196/37365 (PMC9246088; doi:10.2196/37365)
Supplement: Multimedia Appendix 2 [file medinform_v10i6e37365_app2.docx]

**Appendix 2: Interrater agreement matrices for study selection steps.**

**Title and abstract screening**

|  | | **Reviewer 1 (HA)** | | |
| --- | --- | --- | --- | --- |
|  |  | **Include** | **Exclude** | **Total** |
| **Reviewer 2 (ZS)** | **Include** | **49** | **0** | **49** |
|  | **Exclude** | **9** | **209** | **218** |
|  | **Total** | **58** | **209** | **267** |
