# Supplementary material for: Combating COVID-19 Using Generative Adversarial Networks and Artificial Intelligence for Medical Images: Scoping Review
Source: JMIR Med Inform. 2022 Jun 29;10(6):e37365. doi: 10.2196/37365 (PMC9246088; doi:10.2196/37365)
Supplement: Multimedia Appendix 3 [file medinform_v10i6e37365_app3.docx]

**Appendix 3: Data extraction form**

| **Concept** | **Definition** |
| --- | --- |
| **Study Characteristics** |  |
| ID | Unique ID assigned to each study |
| Author | The first author of the study. |
| Year | The year in which the study was published |
| Country of publication | Affiliation of the first author of the study. |
| Publication type | Journal or conference or book chapter |
| Conference name | Name of the conference where the study was published |
| Journal name | Name of the journal where the study was published |
| **GANs method** |  |
| Tasks addressed in the study | What are the applications or uses of AI in COVID-19 pandemic (e.g., diagnosis, drug discovery, projection of cases and deaths)? |
| Purpose of using GAN | The branches/areas of that were used (e.g., traditional machine learning, deep learning, natural language processing). |
| Type of GAN | The specific AI models or algorithms that were used (e.g., Decision tree, Random forest, Convolutional neural network). |
| Key changes in GAN | The platform in which the AI technology was implemented (e.g., computers and mobiles). |
| **Dataset** **Characteristics** |  |
| Data modality | The modality of the data used in the study. For example, CT images, MRI, X Ray, or Ultrasound. |
| Number of sources of data | Is the data collected from single or multiple sources? |
| Type of data sources | Public or private |
| Name of the data source if available | The specific name of the dataset or the source (for example, github or Kaggle) |
| Data source (full URL) | Mention full URL of the dataset |
| Dataset size (Number of subjects or individuals) | For how many individuals the data is recorded? |
| Dataset size (number of images) | What is the total number of images in the images |
| Dataet size (after augmentation) | If GANs were used for data augmentation, what is the total number of images after augmentation with GANs |
| Training set size |  |
| Validation set size |  |
| Testing set size |  |
| Type of validation | Did the authors used train-test split or k-fold cross validation |
| **Evaluation** |  |
| Evaluation metrics | Metrics (for example, Accuracy, Precision, Recall, Dice socre, F1 score, Sensitivitiy, Specificity, SSIM, PSNR) |
| Secondary evaluation | Were the results evaluated by radiologist? |
| How many evaluators? | Two or more evaluators evaluated the results? |
| Code available | Mention the link if the code is made public (for example, on Github?) |
| **Concept** | **Definition** |
| **Study Characteristics** |  |
| ID | Unique ID assigned to each study |
| Author | The first author of the study. |
| Year | The year in which the study was published |
| Country of publication | Affiliation of the first author of the study. |
| Publication type | Journal or conference or book chapter |
| Conference name | Name of the conference where the study was published |
| Journal name | Name of the journal where the study was published |
| **GANs method** |  |
| Tasks addressed in the study | What are the applications or uses of AI in COVID-19 pandemic (e.g., diagnosis, drug discovery, projection of cases and deaths)? |
| Purpose of using GAN | The branches/areas of that were used (e.g., traditional machine learning, deep learning, natural language processing). |
| Purpose of using GAN | What was the specific purpose of using GAN (e.g., for data generation, segmentation, noise removal, segmentation, etc)? |
| Type of GAN | What was the architecture of GAN that was used (e.g., cycleGAN, conditional GAN, Deep Convolutional GAN)? |
|  |  |
| **Dataset** **Characteristics** |  |
| Data modality | The modality of the data used in the study. For example, CT images, MRI, X Ray, or Ultrasound. |
| Number of sources of data | Is the data collected from single or multiple sources? |
| Type of data sources | Public or private |
| Name of the data source if available | The specific name of the dataset or the source (for example, github or Kaggle) |
| Data source (full URL) | Mention full URL of the dataset |
| Dataset size (Number of subjects or individuals) | For how many individuals the data is recorded? |
| Dataset size (number of images) | What is the total number of images in the images |
| Dataet size (after augmentation) | If GANs were used for data augmentation, what is the total number of images after augmentation with GANs |
| Training set size | Number of images used in training data |
| Validation set size | Number of images used in validation data |
| Testing set size | Number of images in test data |
| Type of validation | Did the authors used train-test split or k-fold cross validation |
| **Evaluation** |  |
| Evaluation metrics | Metrics (for example, Accuracy, Precision, Recall, Dice score, F1 score, Sensitivity, Specificity, SSIM, PSNR) |
| Secondary evaluation | Were the results evaluated by radiologist? |
| How many evaluators? | Two or more evaluators evaluated the results? |
| Code available | Mention the link if the code is made public (for example, on Github?) |
